# Testosterone and cortisol concentrations vary with reproductive status in wild female red deer

**DOI:** 10.1002/ece3.1945

**Published:** 2016-01-25

**Authors:** Alyson T. Pavitt, Josephine M. Pemberton, Loeske E. B. Kruuk, Craig A. Walling

**Affiliations:** ^1^ Institute of Evolutionary BiologySchool of Biological SciencesUniversity of EdinburghEdinburghEH9 3FLUK; ^2^ Division of Evolution, Ecology & GeneticsResearch School of BiologyThe Australian National UniversityCanberraACT2601Australia

**Keywords:** Lactating, noninvasive, offspring sex, pregnancy, steroids

## Abstract

Although hormones are key regulators of many fitness and life history traits, the causes of individual level variation in hormones, particularly in wild systems, remain understudied. Whilst we know that androgen and glucocorticoid levels vary within and among individuals in mammalian populations, how this relates to key reproductive processes such as gestation and lactation, and their effects on a female's measurable hormone levels are poorly understood in wild systems. Using fecal samples collected from females in a wild red deer population between 2001 and 2013, we explore how fecal androgen (FAM) and cortisol (FCM) metabolite concentrations change with age and season, and how individual differences relate to variation in reproductive state. Both FAM and FCM levels increase toward parturition, although this only affects FCM levels in older females. FCM levels are also higher when females suckle a male rather than a female calf, possibly due to the higher energetic costs of raising a son. This illustrates the importance of accounting for a female's life history and current reproductive status, as well as temporal variation, when examining individual differences in hormone levels. We discuss these findings in relation to other studies of mammalian systems and in particular to the relatively scarce information on variation in natural levels of hormones in wild populations.

## Introduction

Concentrations of steroid hormones are known to vary extensively both within and between individuals in a population (Williams 2008), yet despite being key regulators of many fitness and life history traits, how and why this variation arises remains understudied in the wild. In the analyses presented here, we explore individual‐level variation in concentrations of female androgens and glucocorticoids within a wild population of red deer (*Cervus elaphus*), and test for associations with season and individual reproductive state. Red deer are seasonal breeders, with females giving birth May–July in the Northern hemisphere. Reproduction (particularly lactation) is a large energetic investment for females (Clutton‐Brock et al. 1983; Catchpole et al. 2004), who, under poor environmental conditions, often have barren (“yeld”) years between calves. This variation allows for investigation of natural endocrine differences between females in different reproductive states.

Whilst androgens (predominantly testosterone) are commonly thought of as male hormones, they are also important in females, where they are secreted in smaller concentrations by the ovaries and adrenal glands (Squires 2003). The fitness and life history effects of high testosterone levels in females of polygynous species are generally negative, although it should be noted that these studies are currently dominated by captive and experimental avian studies. For example, high testosterone female birds show delayed reproduction (Gerlach and Ketterson 2013) and increased immunosuppression (Zysling et al. 2006), whilst female behavior is altered, and survival ultimately reduced, in bank voles with experimentally elevated testosterone (Taitt and Krebs 1982). Data on the extent of variation in testosterone levels, and their life history implications, from wild populations in natural environments are much scarcer.

Although concentrations of testosterone in females tend to be lower than those in males, they do not remain consistent within individual females across their lifetime, but may vary with both age, and time of year (e.g. von Engelhardt et al. 2000). Testosterone levels typically tend to decrease within individuals of both sexes as they age, due to decreased functionality of the hypothalamic‐pituitary‐gonadal axis (Flood et al. 1995). Mean testosterone may also decrease amongst older age classes at the population level due to the loss of high testosterone phenotypes (i.e. “selective disappearance” following van de Pol and Verhulst 2006) if these are at higher risk of early mortality (Taitt and Krebs 1982).

Within a year, temporal variation in testosterone levels often correlates with a number of factors, including seasonal variation in social interactions (von Engelhardt et al. 2000), physiological state, and photoperiod (Davies et al. 1999). Female testosterone increases toward the end of gestation in a number of mammals including humans (Bammann et al. 1980), and domestic livestock (Gaiani et al. 1984; Silberzahn et al. 1984). This has been attributed to both greater hormone secretion (Bammann et al. 1980; Silberzahn et al. 1984), and reduced clearance (Bammann et al. 1980) during this period in captive systems, but remains underexplored in the wild. Lactation may also have an effect, as testosterone levels are known to be lower in humans during lactation than nonlactation (Alder et al. 1986), possibly as a result of suppressed ovarian activity during this time (Howie et al. 1981). Based on this, females in our red deer study population might be expected to have peak androgen levels during May–June when most give birth (Clutton‐Brock et al. 1982). In seasonally reproducing species, environmental changes are usually closely associated with reproductive cycles, making it difficult to differentiate between effects of the two. Here, by specifically testing for links between female steroid hormone levels and pregnancy and lactation, we are able to explore both reproductive state, and any remaining seasonal variation after correcting for changes during the reproductive cycle in red deer.

The second hormone that we consider is cortisol, the primary circulating glucocorticoid in red deer. Whilst an acute increase in circulating levels enables an individual to deal with short‐term stressors (e.g. Wingfield 2003), chronic secretion can have negative implications for fitness (Romero and Butler 2007). As with androgens, glucocorticoids are also known to vary temporally with both age and season, although this is poorly understood in the wild where there may be important fitness implications. Animals often exhibit increased glucocorticoid levels with age due to the physiological effects of aging. Aging may directly influence the secretion of glucocorticoids by reducing the functionality of the endocrine system (e.g. Sapolsky 1991; Thompson et al. 2010), or indirectly through changes, such as reduced condition, which put increasing stress on the body. Given that condition declines from middle‐age onwards in red deer hinds (Clutton‐Brock et al. 1982; Nussey et al. 2009), female cortisol levels in our study system might therefore be expected to increase with age. There is, however, also evidence of females in some systems, including wild seabirds (Heidinger et al. 2010), and humans (Otte et al. 2005), showing reduced glucocorticoid levels as individuals age. This is thought to be due to greater suppression of the stress response during pregnancy amongst older females (see Heidinger et al. 2010 for discussion). Such conflicting conclusions indicate the need for more good empirical data from wild systems.

Where they occur, seasonal glucocorticoid cycles are likely to be closely linked to seasonal variation in stressors such as climatic conditions (Huber et al. 2003a; Weingrill et al. 2004) or variation in reproductive state. Given that glucocorticoid concentrations increase toward the end of gestation across a range of mammals (Cavigelli 1999; Obel et al. 2005), seasonal levels of cortisol might be expected to increase throughout spring and peak May–June (during the calving season) within this study population. Despite the additional stress associated with early lactation, previous studies have found little difference between lactating and nonlactating females (Cook 1997), presumably due to suppression of the acute stress response (Rushen et al. 1995). Variation may, however, still arise between lactating individuals incurring different energetic costs (Cook 1997 and references therein, Hill et al. 2003).

There is some evidence to suggest that both hormone groups may remain consistent amongst individuals across periods of time, with males showing repeatability across days (fecal testosterone metabolites in sheep: Pelletier et al. 2003), months (fecal androgen and glucocorticoid metabolites in geese: Kralj‐Fisher et al. 2007) and years (fecal cortisol but NOT androgen metabolties in red deer: Pavitt et al. 2015). Whilst these studies have primarily focussed on repeatability in males, While et al. (2010) also show circulating testosterone levels to be repeatable in both sexes of the lizard *Egernia whitii* over a several month time period. More work is needed to test whether this among‐individual variation persists in females over longer periods of time, or occurs in other species.

In this study, we address the lack of information on individual variation in hormone levels of wild female mammals by investigating female hormone levels in a wild population of red deer. Specifically, we aimed to determine if females had consistent hormone phenotypes across time, which may have long term implications for their fitness, or if hormone levels were more strongly determined by an individual's current environment and body condition. We explored these local environmental effects by testing whether FAM and FCM levels changed with age and season, and, if seasonal cycles were present, whether they were entirely explained by changes to female reproductive status. The red deer on the Isle of Rum provide an excellent study population to examine these questions in the wild because of the extensive, individual‐based data that have been collected on this system since 1972 (Clutton‐Brock et al. 1982).

## Methods

### Fecal sample collection

Fecal samples were collected from 200 identifiable wild female red deer in the North Block study area of the Isle of Rum National Nature Reserve, Scotland (see Clutton‐Brock et al. 1982 for full description of the study population and site) between 2001 and 2013. Fresh fecal samples were collected within 5 min of witnessing defecation from identifiable individuals. They were stored at −20°C in a field freezer (mean time from collection to freezing: 103.1 min ± 4.0 SE), before being packed in ice and returned to laboratory freezers where they were kept at −20°C until extraction.

### Fecal hormone extraction

Individual fecal samples (806 samples from 200 individuals) were fully defrosted and homogenized to evenly distribute hormones throughout the feces. Once homogenized, 0.5 g of wet sample was extracted with 5 mL of methanol (90%), gently shaken (overnight at 20°C) and centrifuged (20 min at 652 g), after which 1 mL of the resulting supernatant was transferred to a clean tube and stored at −20°C until assay. We used wet, rather than dry, extraction methods because the literature suggests that drying samples first was unlikely to improve the extraction procedure, particularly given the samples used in our study were large and collected almost immediately after defecation (Palme and Touma 2013).

### Fecal hormone enzyme immunoassays (EIA)

FAM and FCM concentrations were measured using group‐specific epiandrosterone (17‐oxo‐androgen) and 11‐oxoetiocholanolone EIAs respectively. Both immunoassays have previously been validated for red deer (Huber et al. 2003b; Pavitt et al. 2015), and were carried out following established methods (Palme and Mostl 1994). The cross‐reactivities have previously been reported for both epiandrosterone (Palme and Mostl 1994) and 11‐oxoetiocholanolone (Huber et al. 2003b) antibodies. Serial dilutions of 24 pooled samples showed high parallelism with the standard curve in both hormone groups (*P* < 0.001), which had limits of detection (LOD) of 0.89 ng/g wet weight (WW) and 3.51 ng/g WW for the FAM and FCM assays respectively. The intra‐ and interassay coefficients of variance (CV) were calculated at 4.26% and 20.64% for FAM and 4.31% and 18.65% for the FCM assay based on biological controls from pooled fecal samples. Several assay plates were run per day and given that previous studies have found assay date to account for significant variation between samples (e.g. Graham et al. 2010; Pavitt et al. 2014, 2015), the mean within‐day inter‐assay CV was also calculated. This gave a mean within‐day interassay CV of 12.46% (±1.80 SE) for FAM and 15.02% (±3.72 SE) for FCM. From the original 688 FAM measures and 804 FCM measures, 11 FAM and 15 FCM were removed due to low repeatability of concentrations measured (CV > 10%). A further 39 FAM and 24 FCM were removed for falling below the LOD (removal of these <LOD measures did not affect the results of the model, see Table S1 for details).

### Statistical analysis

A bivariate (i.e. “multiresponse”) mixed model was fitted to the data in ASReml‐R ver. 3.0.3 (package: asreml, Butler 2009) to explore potential causes of (co)variation in female FAM and FCM levels, using the model structure:

FAM, FCM ~ fixed_effects + (individual ID)          + (year) + (residuals).

The fixed and random (in parentheses) effects are discussed below. Both hormone measures were log‐transformed to normalize the residuals, and all continuous fixed effects were mean‐centred. A total of 638 FAM measures (from 192 females) and 765 FCM measures (from 194 females) were included in this analysis (see Fig. S1 for distribution of the number of times individuals were sampled).

### Fixed effects

We fitted models containing the following fixed effects for both hormone groups:

*Age*: Continuous variable measured in years, tested as quadratic as well as linear.

*Age at last sampling*: Continuous variable measured in years and used to test for the selective disappearance of individuals with particular hormone phenotypes (i.e. whether a late age at last sampling for an individual is associated with relatively high or low hormone values across all measures); such selective disappearance may influence any apparent age‐related trends (van de Pol and Verhulst 2006).

*Sample month*: A 12‐level factor for the month in which the fecal sample was collected.

*Assay date*: An eight‐level factor for the date the sample was assayed.

*Pregnancy status*: A two‐level factor for whether a female (1) was not pregnant or was sampled in early pregnancy (defined as up to 118 days after estimated conception date) versus (2) was sampled during late pregnancy (defined as the period of time from 119 days after estimated conception date until parturition, which is an average of 235 days after estimated conception; Clements et al. 2011). In preliminary analyses, there was no significant difference between samples from non‐pregnant and early pregnancy females, and so these were combined into a single factor level.

*Lactation status*: A three‐level factor for whether a female was (1) suckling a male calf, (2) suckling a female calf, or (3) not lactating at the time the sample was collected. Females were considered to be lactating if they were sampled before April with a live calf (<1 year old) from the previous calving season. If females had not calved during the previous calving season, or had given birth to a calf that had died before the date of sampling, the female was considered to be “not lactating”.

*Sample time to freezing*: Continuous variable measuring the time (in minutes) from fecal collection to freezing (in a −20°C field freezer). This was tested because hormone concentrations are known to change over relatively short periods of time in samples kept at ambient temperature (Mostl et al. 1999). Collection and freezing times were not recorded for 203 samples, and whilst initial analyses originally omitted these samples, they were included in the final model with the mean time at ambient (calculated from all samples which did have a measure) to avoid the loss of data. This gave the model greater statistical power and did not cause the trends in the main findings to change (see Table S2 for model output excluding samples with no record of time at ambient temperature).

### Random effects

The model also included individual identity (*n* = 200), sampling year (*n* = 13), and an unexplained residual (error) term as random effects for both traits. Inclusion of these random effects allowed estimation of the among‐individual variance (variance due to individual identity), the among‐year variance (variance due to year) and the within‐individual (residual or error) variance.

The use of bivariate models allows the estimation of the covariance between FAM and FCM concentrations at all three levels (among‐individual, year and within‐individual). After testing the variance components, however, only the within‐individual (residual) component explained significant variance and so the only covariance estimated in the final model was between FAM and FCM at the within‐individual level. To ensure that the lack of among‐individual variance in FAM and FCM levels was not an artefact of between‐sample covariance declining over time, the autocorrelation between samples was also tested (see supporting information 1.1 for full methods). Correcting for temporal autocorrelation did not, however, significantly improve the model (see Table S3 for model details) and so was not included in the final analyses.

Fixed effects were tested for significance using incremental Wald tests, and the optimal model was accepted when all remaining fixed effects were significant at *P* < 0.05. The variances and covariances were tested for statistical significance using likelihood ratio tests (assuming a chi‐squared distribution with 1 degree of freedom) comparing the full model with models where each variance (or covariance) term was fixed to 0 in turn. The total variance (*r* ^2^) accounted for by the fixed effects was also estimated individually for FAM and FCM levels from linear mixed effect models in R 3.1.1 (package: lme4, Bates et al. 2011) following methods in Nakagawa and Schielzeth (2013).

## Results

Both fecal androgens and cortisol metabolite concentrations showed substantial variation between samples. FAM concentrations ranged from 1.02 to 311.70 ng/g WW (mean: 22.93 ng/g WW ± 1.16 SE) and FCM concentrations from 4.33 to 20720.00 ng/g WW (mean: 106.35 ng/g WW ± 31.87 SE).

Variation in laboratory and collection conditions had small but significant effects on both hormone groups. Both FAM and FCM levels varied depending on the date they were assayed (*P* < 0.001); FAM levels also increased by 1.36% for each hour taken between sample collection and freezing (*P* = 0.007), although time to freezing did not affect FCM levels (*P* = 0.370) (Table 1; see supporting information 1.2 for discussion).

**Table 1:**
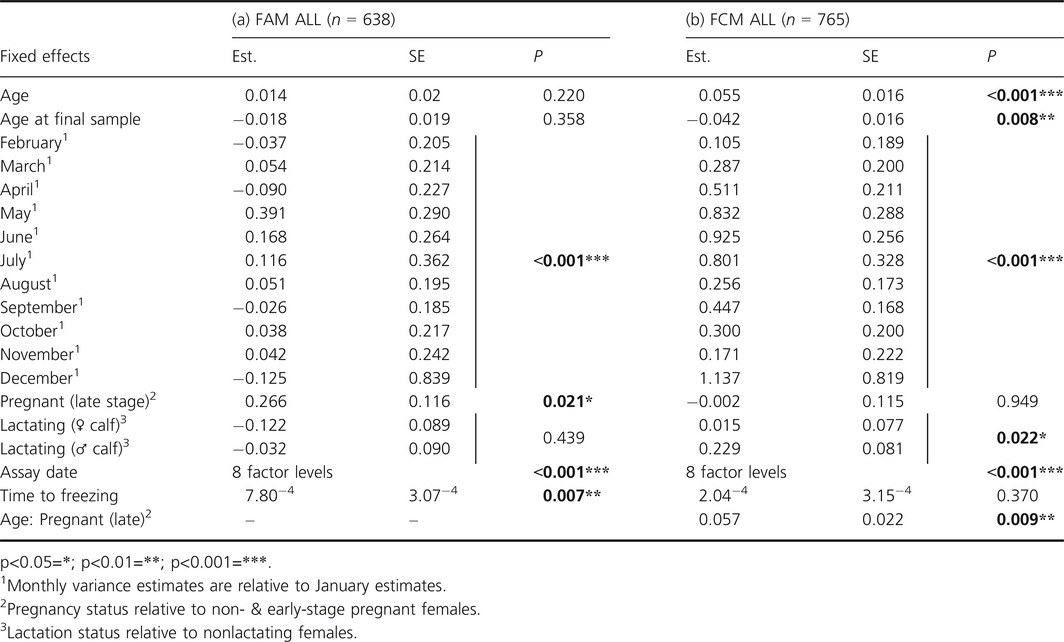
Fixed effects from bivariate mixed effect models examining the main effects of age, season and reproductive state on interindividual variation in female log‐transformed (a) FAM and (b) FCM concentrations

**Figure 1:**
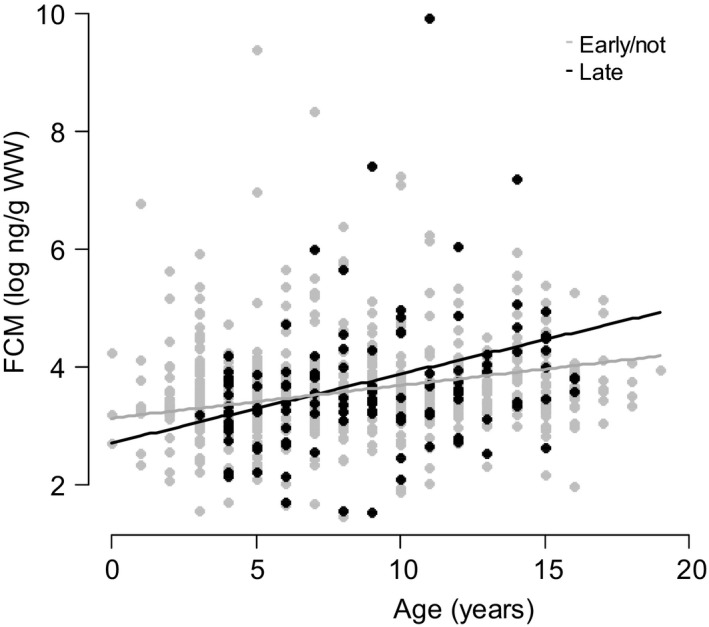
Variation in log transformed fecal cortisol metabolite (FCM) concentrations with age amongst females which were not pregnant or sampled during early pregnancy (gray) and those sampled in late pregnancy (black), showing the raw data points plotted beneath the FCM predictions estimated from a model correcting for all significant fixed effects shown in Table 1b.

**Figure 2:**
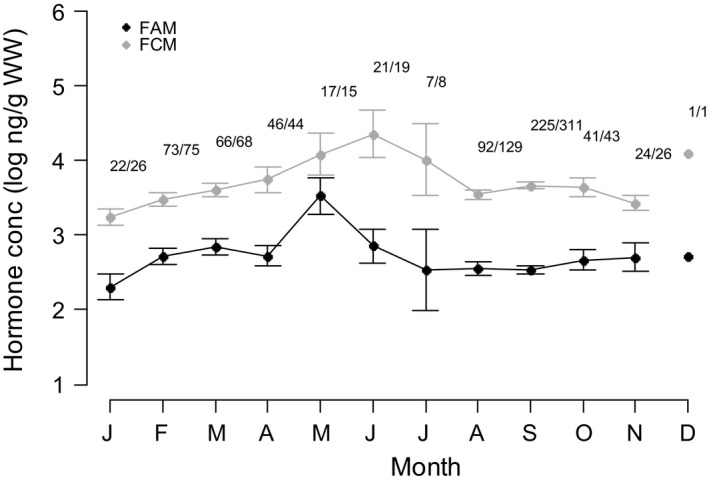
Variation in log‐transformed faecal androgen metabolite (FAM; black) and fecal cortisol metabolite (FCM; gray) concentrations with month. Points represent monthly averages ± standard errors of raw data values. Numbers represent monthly sample sizes for FAM/FCM respectively. Only one sample was collected in December and so no estimate of error could be provided.

After accounting for variation due to assay date and the time from sample collection to freezing, concentrations of FCM but not FAM showed significant variation with age (FAM: *P* = 0.220, FCM: *P* < 0.001; Table 1), and with age of final sample (FAM: *P* = 0.358, FCM: *P* = 0.008; Table 1). FCM levels increased linearly as females aged (Fig. 1), with 10 year old females having 7.02% higher (log) FCM levels than 5 year olds. At the same time, there was a *decrease* in FCM levels with age at final sampling. This indicates that FCM increased with age within individuals, but that females sampled in the older age groups were more likely to have lower FCM levels. Both hormones did, however, show similar patterns of monthly variation (*P* < 0.001; Table 1; Fig. 2). FAM levels spiked in May (a 15.18% increase from April), whilst FCM increased through May to June (a 9.46% increase from April, and a 24.73% increase from January), coinciding with the calving season and fell again in July.

In addition to age‐ and season‐related effects, we also explored the relationships between female reproductive status and fecal hormone concentrations. Both hormones varied with pregnancy status (Table 1). Levels of (log) FAM were 8.66% higher during late pregnancy compared to females who were in early pregnancy or not pregnant at all (Table 1; *P* = 0.021). Late stage pregnancy was also associated with higher FCM levels but only in older age groups (*P* = 0.009; Table 1; Fig. 1). In addition, FCM concentrations also varied depending on the female's lactation status at the time of sampling (*P* = 0.022; Table 1). (Log) FCM levels were 5.71% higher in females suckling a son versus a daughter, and 6.20% higher compared to those not lactating at all. Females suckling a daughter did not appear to differ from nonlactating females (0.47% difference). Overall, the fixed effects explained 39.27% of the variance seen in FAM levels, and 13.91% of that seen in FCM levels based on estimated *r* ^2^ values from linear mixed models.

After accounting for the effects of age, season and reproductive state there was no evidence of either among‐individual or among‐year variation for either hormone (Table 2). There was significant within‐individual variance for both hormones, however the within‐individual covariance between FAM and FCM was nonsignificant (*P* = 0.350; Table 2). This indicates that the relative difference between levels of FAM and FCM did not remain consistent within an individual female between samples.

**Table 2:** Estimated (co)variance components from a bivariate mixed effects model (fixed effects presented in Table 1) for fecal androgen metabolites (FAM) and fecal cortisol metabolites (FCM) at (a) among‐individual, (b) among‐year, and (c) within‐individual (residual) levels. Variances are presented on the diagonal, covariances below the diagonal, and correlations above the diagonal. Standard errors are in parentheses

(a) Among‐individual			(b) Among‐year			(c) Within‐individual		
	FAM	FCM		FAM	FCM		FAM	FCM
FAM	0 (NA)		FAM	0.002 (0.003)		FAM	**0.660 (0.038)**	0.031 (0.034)
FCM		0.002 (0.013)	FCM		<0.001 (0.001)	FCM	0.025 (0.027)	**0.637 (0.035)**

Shaded cells indicate values that were not estimated as a result of non‐significant variance estimates. Statistically significant (*P* < 0.05) variances and covariances are in bold.

## Discussion

This study explores correlates of variation in androgen and glucocorticoid levels, two hormone groups known to play key regulatory roles in many fitness and life history traits. Using noninvasively obtained (fecal) measures of androgen and cortisol metabolites collected repeatedly from known individuals, we quantified hormone variation both within and among individuals in a wild population of red deer. There was no evidence of among‐individual variance for either hormone group, nor were there any consistent among‐year differences. This suggests that an individual female's hormone levels were more determined by a combination of their present state and immediate environment than by a consistent hormone phenotype. The exact associations with state and environment did, however, differ between the hormone groups. Both showed similar seasonal cycles, peaking around the spring/summer calving season, but only FCM levels changed with age. FAM and FCM levels also varied with the female's pregnancy status at the time of sampling, with an additional association between offspring sex and FCM levels during lactation.

Despite evidence for short‐term repeatability of testosterone levels in other species (While et al. 2010), we found no evidence of among‐individual variance of FAM levels amongst individual females in this study. This concurs both with previous results from male red deer in this population (Pavitt et al. 2015), and with evidence from other studies showing endocrine measures in other species to become less repeatable when considered over increased time periods (e.g. Boulton et al. 2015). Our results may therefore differ from other studies because we collected samples over several years, whilst previous studies have been assessed repeatability within shorter time periods of days (e.g. Pelletier et al. 2003) or at most months (e.g. While et al. 2010). The lack of temporal autocorrelation (Table S3), however, suggests that there was no trend in the correlation over time (i.e. the correlation between samples did not decrease as time increased). This, coupled with the fact that samples collected 3 days apart had a correlation of <0.01 (Table S3b), suggests that the lack of repeatability in these data are not simply a result of large time differences between some samples from the same individual. FAM levels in this population, therefore, appear to be more influenced by the female's environment and state around the time of sampling than consistent difference between individuals. These environmental effects are discussed below.

There was no link between age and FAM levels at either the individual or population level. This suggests no consistent within‐individual change in FAM with age, nor any evidence of particular FAM phenotypes being selectively lost from the population in older age classes. FAM levels did, however, show substantial seasonal variation, peaking in May to coincide with the start of the calving season. This seasonal cycle remains despite accounting for the female's reproductive state in the model, suggesting effects beyond those of the reproductive cycle. This also fits with a more general trend toward higher FAM concentrations during late gestation (Table 1), and is consistent with findings from domestic livestock (Gaiani et al. 1984; Silberzahn et al. 1984) where late pregnancy is associated with an increase in steroid production (Silberzahn et al. 1984). The absence of any lactation effect on FAM concentrations indicates that, in this population, the physiological changes associated with lactation are not reflected in androgen levels.

In direct contrast with male red deer in our study population (Pavitt et al. 2015), we found no among‐individual variance in FCM levels in females. When formally tested, this between‐sex difference in FAM repeatability was found to be statistically significant (*χ* ^2^_(1)_ = 3.583, *P* = 0.007; Table S4). This indicates that female FCM levels are significantly less consistent at the individual‐level than male levels, potentially due to temporal changes in the sensitivity of the stress response at different stages of pregnancy and lactation (Obel et al. 2005). As with the lack of a consistent FAM phenotype, the result suggests that FCM levels in female deer are more influenced by changes in local environment and state than consistent individual differences.

When testing the relationship between state and hormone levels, there were clear age‐related trends in FCM concentrations. The within‐individual increase in FCM levels with age is consistent with the aging effect seen in FCM levels within males of this population (Pavitt et al. 2015). This may arise due to age related desensitization of the cortisol stress response (Thompson et al. 2010), or as a consequence of older females being in poorer condition, as both disease and general senescence are associated with higher glucocorticoid levels (Reeder and Kramer 2005). Whilst FCM appears to increase within individuals as they age, individuals who reached an older age of last sample had lower FCM levels on average. This suggests selective disappearance of high FCM individuals, although the small effect size (a 1.3% decrease in (log) FCM levels per year; Table 1) indicates that the effect is a weak one. Given that high cortisol levels may be associated with reduced fitness (Romero and Butler 2007), the selective disappearance of high FCM females with age could be indicative of reduced lifespans, although the dataset was collected too recently to fully test this as most individuals were still alive. It is also possible that this trend may arise as a consequence of sampling bias, i.e. older females with high FCM levels are still alive but are just less likely to be sampled for some as yet unknown reason.

Despite no consistent variation between years, there were clear seasonal cycles within years, with FCM peaking in May‐June to coincide with the calving season even after correcting for any variation associated with reproductive state. Inherent seasonal cycling in baseline cortisol levels could be an adaptive response to regular and predictable changes in stressor intensity associated with the calving season, but independent of the female's present reproductive state. The Preparative Hypothesis (Romero 2002) proposes that baseline glucocorticoids may have evolved to increase during stressful periods of the year (such as the reproductive season), thus enabling the body to better respond to those stressors should they arise.

Whilst there was no overall variation in FCM levels with pregnancy status, older females had significantly higher levels during late gestation than non‐pregnant females of a similar age (Fig. 1). This is likely to occur due to pregnancy‐linked stressors (which arise as a consequence of the high cost of reproduction, Catchpole et al. 2004) being amplified by the age‐related changes in the stress response (see above). In concurrence with previous studies (Hill et al. 2003), there was also no overall difference in FCM levels between lactating and non‐lactating females, however females suckling a son had much higher FCM levels than females who had a daughter, or who were not lactating at all. These high FCM levels suggest an overall increase in stress levels when rearing a son versus rearing a daughter (or not having a calf at all), possibly as a result of sons being more costly to rear than daughters (Clutton‐Brock et al. 1981) because of their greater body weight (Clutton‐Brock et al. 1982) and higher milk demands (Landete‐Castillejos et al. 2005).

The absence of any among‐individual variance for FAM and FCM levels indicates that there were no consistent differences between individuals (i.e. no consistent hormone phenotypes), and therefore the covariance between them could not be estimated. This, coupled with the nonsignificant within‐individual covariance between FAM and FCM levels, indicated the absence of any consistent relationship between the two hormone groups in this population. Consequently, females with high concentrations of one hormone were no more likely to have higher concentrations of the other. There was also no evidence of a relationship between these two hormone groups for males in this population (Pavitt et al. 2015). This suggests that, contrary with some studies which show stress (and associated high glucocorticoids) to inhibit testosterone secretion (see Perez‐Rodriguez et al. 2006), concentrations of FAM in these female red deer do not exhibit predictable changes with stress.

In summary, both androgen and cortisol metabolite concentrations varied considerably over time and with a female's reproductive status. Only FCM levels varied with age, but both FAM and FCM showed pronounced seasonal cycles, with concentrations peaking around the spring calving season. Reproductive status was also found to be important in explaining variation between samples. Levels of both hormone groups were higher during late pregnancy, although FCM levels only showed this trend in older females, possibly due to a combination of pregnancy‐linked stressors and physiological aging of the cortisol response. Lactating FCM concentrations were also higher when mothers were suckling a male calf, possibly due to the higher energetic cost of raising a son. After accounting for age, season, and reproductive status, neither hormone group showed any among‐individual or among‐year variance.

## Ethical Approval

This article does not contain experimental manipulation of the study animals by any of the authors; all samples and observations were collected noninvasively.

## Conflict of Interest

None declared.

## Supporting information


**Appendix S1.** Supporting text.
**Figure S1.** Distribution of number of faecal samples collected per individual female for (a) FAM (*n* = 638 samples from 192 females) and (b) FCM (*n* = 764 samples from 194 females).
**Table S1.** In the main text, we report analyses of data in which values of FAM or FCM that fell below the limits of detection (LOD) had been removed (39 FAM and 24 FCM). (a) Fixed effects from the multivariate mixed effects models with hormone measures falling below the LOD included in the analyses (see Table S1b for (co)variance components). (b) Estimates of variance (diagonal), covariance (below diagonal) and correlation (above diagonal) components from the multivariate mixed effects models with hormone measures falling below the LOD included in the analyses (see Table S1a for fixed effects).
**Table S2.** Fixed effects from bivariate mixed models examining the main effects of season and state on inter‐individual variation in female (a) FAM and (b) FCM concentrations, considering only those samples with known collection and freezing times.
**Table S3.** Outputs from linear mixed models examining the main effects of season and state on inter‐individual variation in female FAM and FCM concentrations after correcting for temporal autocorrelation. (a) Fixed effects from the linear mixed models. (b) Temporal autocorrelation structure and *P*‐values from nested anova tests comparing models with and without the autocorrelation structure.
**Table S4.** Bivariate mixed model estimating (a) among‐individual, and (b) within‐individual variance for male (*n* = 178) and female (*n* = 768) FCM concentrations (after being standardised for sex‐specific variance).
