# Supplementary material for: Testosterone and cortisol concentrations vary with reproductive status in wild female red deer
Source: Ecol Evol. 2016 Jan 25;6(4):1163–72. doi: 10.1002/ece3.1945 (PMC4761757; doi:10.1002/ece3.1945)
Supplement: Supplementary file 1 — Appendix S1. Supporting text. Figure S1. Distribution of number of faecal samples collected per individual female for (a) FAM (n = 638 samples from 192 females) and (b) FCM (n = 764 samples from 194 females). Table S1. In the main text, we report analyses of data in which values of FAM or FCM that fell below the limits of detection (LOD) had been removed (39 FAM and 24 FCM). (a) Fixed effects from the multivariate mixed effects models with hormone measures falling below the LOD included in the analyses (see Table S1b for (co)variance components). (b) Estimates of variance (diagonal), covariance (below diagonal) and correlation (above diagonal) components from the multivariate mixed effects models with hormone measures falling below the LOD included in the analyses (see Table S1a for fixed effects). Table S2. Fixed effects from bivariate mixed models examining the main effects of season and state on inter‐individual variation in female (a) FAM and (b) FCM concentrations, considering only those samples with known collection and freezing times. Table S3. Outputs from linear mixed models examining the main effects of season and state on inter‐individual variation in female FAM and FCM concentrations after correcting for temporal autocorrelation. (a) Fixed effects from the linear mixed models. (b) Temporal autocorrelation structure and P‐values from nested anova tests comparing models with and without the autocorrelation structure. Table S4. Bivariate mixed model estimating (a) among‐individual, and (b) within‐individual variance for male (n = 178) and female (n = 768) FCM concentrations (after being standardised for sex‐specific variance). [file ECE3-6-1163-s001.docx]

**Supporting material for: Testosterone and cortisol concentrations vary with reproductive status in wild female red deer**

Alyson T Pavitt, Josephine M Pemberton, Loeske EB Kruuk, and Craig A Walling

**1. SUPPORTING TEXT**

**1.1: TEMPORAL AUTOCORRELATION METHODS**

To test that the lack of among-individual variance in FAM and FCM levels was not due to the between-sample covariance declining over time, univariate linear mixed effect models were also fitted individually to the data on each hormone (with the same fixed effects) in R 3.1.1 (package: nlme, Pinheiro et al., 2014) with a correlation structure included. Where individuals were sampled more than once on the same day (n=12 instances), the mean value for that day was used. This enabled us to test for autocorrelation between samples collected on different days at the individual level. An ANOVA was used to test models with and without the correlation structure included, and found the models correcting for temporal autocorrelation between days were not significantly better than ones which did not (FAM: p=0.965; FCM: p=0.555; see Table s3 for model details). This indicated that the covariance between samples did not decline over time, and therefore this was not the cause of the lack of among-individual variance. An autocorrelation structure was therefore not fitted to the final bivariate model.

**1.2: DISCUSSION OF SAMPLING EFFECTS**

Given the high bacterial load in faeces, faecal steroids are generally considered less stable than those in blood or urine samples (Whitten et al., 1998). If not accounted for, this can introduce considerable noise and error into measures of faecal hormone concentration. The increase in FAM (faecal androgen metabolite) levels with time to freezing (i.e. time at ambient temperature) suggests that faecal bacteria were synthesising androgens from other faecal steroids. Although the effect size was small (1.36% change in log FAM levels per hour), the statistical significance indicates that concentrations of the androgens measured in this assay (i.e. captured by the antibody) and their precursors cannot be considered entirely stable, even within hours of defecation, and should be frozen at -20^o^c as soon as possible. By contrast, FCM (faecal cortisol metabolite) concentrations remained more stable across time, indicating that the cortisol metabolites were less prone to the effects of bacterial synthesis during the first day of faecal collection. There was, nevertheless, a non-significant trend towards higher FCM levels with increased time at ambient temperature, indicating that care should still be taken to minimise time between defecation and freezing. In agreement with previous studies, assay date also explained significant variation between samples (Pavitt et al., 2014, Graham et al., 2010). This could be a consequence of variation in laboratory conditions (e.g. lab temperature) between days (Graham et al., 2010), but the exact causes of this variation are currently unknown.

GRAHAM, A., HAYWARD, A., WATT, K., PILKINGTON, J., PEMBERTON, J. & NUSSEY, D. 2010. Fitness correlates of heritable variation in antibody responsiveness in a wild mammal. *Science,* 330**,** 662-665.

PAVITT, A., WALLING, C., PEMBERTON, J. & KRUUK, L. 2014. Causes and consequences of variation in early life testosterone in a wild population of red deer. *Functional Ecology,* 28**,** 1224-1234.

PINHEIRO, J., BATES, D., DEBROY, S. & SARKAR, D. 2014. nlme: linear and nonlinear mixed effects models.

WHITTEN, P., BROCKMAN, D. & STAVISKY, R. 1998. Recent advances in noninvasive techniques to monitor hormone-behavior interactions. *Yearbook of Physical Anthropology,* 41**,** 1-23.

**2. SUPPORTING FIGURES**

**2.1: FIGURE S1**

*Distribution of number of faecal samples collected per individual female for (a) FAM (n=638 samples from 192 females) and (b) FCM (n=764 samples from 194 females)*

******

**3. SUPPORTING TABLES**

**3.1: TABLE S1**

*In the main text, we report analyses of data in which values of FAM or FCM that fell below the limits of detection (LOD) had been removed (39 FAM and 24 FCM). Here we check whether this removal affected the results in any way by repeating the analyses with their values replaced by the LOD value. Tables s1a & b show multivariate mixed effects model estimating the main effects (Table s1a) and (co)variance components (Table s1b) of extrinsic factors on individual-level variation in (a) FAM concentrations, and (b) FCM concentrations when measures falling below the LOD (FAM: n=39; FCM: n=24) were included in the analyses. These <LOD samples were assigned the LOD value (FAM: 0.89 ng/ g faeces; FCM: 3.51 ng/g faeces) because these were the lowest measures that could be reliably estimated from the standard curve. The conclusions are the same.*

***Table s1a:*** *Fixed effects from the multivariate mixed effects models with hormone measures falling below the LOD included in the analyses (see Table s1b for (co)variance components).*

|  | (a) FAM ALL (n=677) | | | | (b) FCM ALL (n=789) | | | |
| --- | --- | --- | --- | --- | --- | --- | --- | --- |
| **FIXED EFFECTS** | Est. | SE | p |  | Est. | SE | p |  |
| Age | 0.000 | 0.020 | 0.220 |  | 0.043 | 0.018 | <0.001 | *** |
| Age at final sample | -0.004 | 0.020 | 0.883 |  | -0.032 | 0.018 | 0.087 | . |
| February ^a^ | -0.060 | 0.215 | <0.001 | *** | 0.026 | 0.203 | <0.001 | *** |
| March ^a^ | 0.061 | 0.226 |  |  | 0.204 | 0.215 |  |  |
| April ^a^ | -0.050 | 0.242 |  |  | 0.442 | 0.230 |  |  |
| May ^a^ | 0.342 | 0.306 |  |  | 0.409 | 0.294 |  |  |
| June ^a^ | 0.264 | 0.282 |  |  | 0.831 | 0.277 |  |  |
| July ^a^ | 0.267 | 0.390 |  |  | 0.827 | 0.360 |  |  |
| August ^a^ | -0.040 | 0.203 |  |  | 0.308 | 0.187 |  |  |
| September ^a^ | -0.016 | 0.193 |  |  | 0.525 | 0.181 |  |  |
| October ^a^ | 0.016 | 0.226 |  |  | 0.386 | 0.217 |  |  |
| November ^a^ | 0.061 | 0.255 |  |  | 0.165 | 0.240 |  |  |
| December ^a^ | -0.218 | 0.912 |  |  | 1.104 | 0.901 |  |  |
| Pregnant (late)^b^ | 0.278 | 0.125 | 0.021 | * | 0.109 | 0.123 | 0.949 |  |
| Lactating (♀ calf)^c^ | -0.036 | 0.095 | 0.936 |  | 0.036 | 0.084 | 0.022 | * |
| Lactating (♂ calf) ^c^ | -0.004 | 0.094 |  |  | 0.242 | 0.088 |  |  |
| Assay date | 8 factor levels | | <0.001 | *** | 8 factor levels | | <0.001 | *** |
| Time to freezing | 0.001 | <0.001 | 0.007 | ** | <0.001 | <0.001 | 0.412 |  |
|  |  |  |  |  |  |  |  |  |
| Age:Pregnant (late)^b^ | - | - |  |  | 0.060 | 0.024 | 0.009 | ** |

^a^ monthly variance estimates are relative to January estimates

^b^ pregnancy status relative to non- & early- pregnant females

^c^ lactation status relative to non-lactating females

***Table s1b:*** *Estimates of variance (diagonal), covariance (below diagonal) and correlation (above diagonal) components from the multivariate mixed effects models with hormone measures falling below the LOD included in the analyses (see Table s1a for fixed effects).*

| **(a) Among-individual** | | |  | **(b) Among-year** | | |  | | **(c) Within-individual** | | | |
| --- | --- | --- | --- | --- | --- | --- | --- | --- | --- | --- | --- | --- |
|  | M | F |  |  | M | F | |  |  | M | F |  |
| M | 0 (NA) |  |  | M | 0.001 (0.002) |  | |  | M | **0.782 (0.047)** | 0.035 (0.035) |  |
| F |  | 0.014 (0.019) |  | F |  | <0.001 (0.001) | |  | F | 0.030 (0.031) | **0.763 (0.043)** |  |

**3.2: TABLE S2**

*Fixed effects from bivariate mixed models examining the main effects of season and state on inter-individual variation in female (a) FAM and (b) FCM concentrations, considering only those samples with known collection and freezing times. For the analyses presented in the main text, missing collection and freezing times were replaced by their mean values, in order to use the information on that sample. The conclusions are the same.*

|  | FAM ALL (n=484) | | | | | FCM ALL (n=576) | | | | |
| --- | --- | --- | --- | --- | --- | --- | --- | --- | --- | --- |
| **FIXED EFFECTS** | Var | SE | z-ratio | p |  | Var | SE | z-ratio | p |  |
| Age | -0.005 | 0.038 | -0.122 | 0.636 |  | 0.033 | 0.031 | 1.053 | 0.012 | * |
| Age of final sample | 0.008 | 0.039 | 0.200 | 0.956 |  | -0.022 | 0.031 | -0.720 | 0.516 |  |
| February ^a^ | -0.225 | 0.244 | -0.924 | 0.001 | ** | -0.025 | 0.245 | -0.104 | <0.001 | *** |
| March ^a^ | -0.096 | 0.254 | -0.378 |  |  | 0.286 | 0.258 | 1.109 |  |  |
| April ^a^ | -0.309 | 0.267 | -1.160 |  |  | 0.581 | 0.269 | 2.161 |  |  |
| May ^a^ | 0.068 | 0.348 | 0.197 |  |  | 0.936 | 0.367 | 2.552 |  |  |
| June ^a^ | -0.091 | 0.318 | -0.285 |  |  | 0.804 | 0.323 | 2.491 |  |  |
| July ^a^ | 0.000 | NA | NA |  |  | 0.776 | 0.851 | 0.912 |  |  |
| August ^a^ | -0.230 | 0.239 | -0.964 |  |  | 0.170 | 0.232 | 0.729 |  |  |
| September ^a^ | -0.177 | 0.226 | -0.780 |  |  | 0.418 | 0.227 | 1.840 |  |  |
| October ^a^ | -0.138 | 0.259 | -0.534 |  |  | 0.335 | 0.259 | 1.294 |  |  |
| November ^a^ | -0.105 | 0.300 | -0.349 |  |  | 0.141 | 0.288 | 0.489 |  |  |
| December ^a^ | 0.000 | NA | NA |  |  | 0.000 | NA | NA |  |  |
| Pregnant (late) ^b^ | 0.259 | 0.123 | 2.098 | 0.060 | . | 0.008 | 0.128 | 0.066 | 0.927 |  |
| Lactating (♀ calf) ^c^ | -0.102 | 0.098 | -1.034 | 0.568 |  | 0.029 | 0.088 | 0.327 | 0.039 | * |
| Lactating (♂ calf) ^c^ | 0.037 | 0.100 | 0.368 |  |  | 0.243 | 0.092 | 2.644 |  |  |
| Assay date (2) ^d^ | -0.855 | 0.231 | -3.701 | <0.001 | *** | -0.078 | 0.140 | -0.557 | <0.001 | *** |
| Assay date (3) ^d^ | -1.835 | 0.225 | -8.140 |  |  | -0.068 | 0.135 | -0.505 |  |  |
| Assay date (4) ^d^ | -0.319 | 0.223 | -1.432 |  |  | 0.462 | 0.133 | 3.479 |  |  |
| Assay date (5) ^d^ | -0.232 | 0.220 | -1.052 |  |  | -0.470 | 0.197 | -2.389 |  |  |
| Assay date (6) ^d^ | -0.175 | 0.227 | -0.771 |  |  | -0.259 | 0.138 | -1.873 |  |  |
| Assay date (7) ^d^ | -0.497 | 0.234 | -2.122 |  |  | -0.207 | 0.114 | -1.811 |  |  |
| Time to freezing | 0.001 | 0.000 | 2.540 | 0.014 | * | 2.04^-04^ | 3.15^-04^ | 0.649 | 0.526 |  |
|  |  |  |  |  |  |  |  |  |  |  |
| Age:Pregnant (late)^b^ | - | - | - |  |  | 0.049 | 0.025 | 1.944 | 0.047 | * |

^a^ monthly variance estimates are relative to January estimates

^b^ pregnancy status relative to non- & early- pregnant females

^c^ lactation status relative to non-lactating females

^d^ assay date variance estimates are relative to the first assay date

**3.3: TABLE S3**

*Outputs from linear mixed models examining the main effects of season and state on inter-individual variation in female FAM and FCM concentrations after correcting for temporal autocorrelation*

***Table s3a****: Fixed effects from the linear mixed models*

|  | FAM ALL (n=611) | | | | FCM ALL (n=735) | | | |
| --- | --- | --- | --- | --- | --- | --- | --- | --- |
| **FIXED EFFECTS** | Est. | SE | p |  | Est. | SE | p |  |
| Age | -0.012 | 0.017 | 0.491 |  | 0.059 | 0.015 | <0.001 | *** |
| Age of final sample | 0.013 | 0.016 | 0.419 |  | -0.046 | 0.015 | 0.002 | ** |
| February ^a^ | -0.086 | 0.215 | <0.001 | *** | 0.063 | 0.200 | <0.001 | *** |
| March ^a^ | 0.030 | 0.225 |  |  | 0.241 | 0.210 |  |  |
| April ^a^ | -0.106 | 0.238 |  |  | 0.484 | 0.223 |  |  |
| May ^a^ | 0.385 | 0.300 |  |  | 0.780 | 0.299 |  |  |
| June ^a^ | 0.176 | 0.273 |  |  | 0.864 | 0.267 |  |  |
| July ^a^ | 0.148 | 0.371 |  |  | 0.758 | 0.338 |  |  |
| August ^a^ | 0.080 | 0.206 |  |  | 0.196 | 0.186 |  |  |
| September ^a^ | -0.030 | 0.196 |  |  | 0.422 | 0.180 |  |  |
| October ^a^ | -0.003 | 0.228 |  |  | 0.250 | 0.213 |  |  |
| November ^a^ | 0.002 | 0.253 |  |  | 0.133 | 0.235 |  |  |
| December ^a^ | -0.194 | 0.851 |  |  | 1.102 | 0.833 |  |  |
| Pregnant (late)^b^ | 0.270 | 0.119 | 0.024 | * | 0.004 | 0.117 | 0.971 |  |
| Lactating (♀ calf)^c^ | -0.105 | 0.092 | 0.253 |  | -0.001 | 0.080 | 0.988 | * |
| Lactating (♂ calf) ^c^ | -0.058 | 0.093 | 0.531 |  | 0.234 | 0.084 | 0.006 |  |
| Assay date | 7 factor levels | | <0.001 | *** | 7 factor levels | | <0.001 | *** |
| Time to freezing | <0.001 | <0.001 | 0.005 | ** | <0.001 | <0.001 | 0.329 |  |
|  |  |  |  |  |  |  |  |  |
| Age:Pregnant (late)^b^ | - | - | - |  | 0.057 | 0.023 | 0.012 | ** |

***Table s3b****: Temporal autocorrelation structure and p-values from nested anova tests comparing models with and without the autocorrelation structure. Phi estimates the strength of the correlation between two samples collected a day apart.*

|  | **Sample size** | **No. of individuals** | **Phi** | **Phi 95% CI** | **p-value** |
| --- | --- | --- | --- | --- | --- |
| FAM | 611 | 187 | 0.174 | 0-1 | 0.965 |
| FCM | 735 | 189 | 0.204 | 0.007-0.898 | 0.555 |

**3.4: TABLE S4**

*Bivariate mixed model estimating (a) among-individual, and (b) within-individual variance for male (n=178) and female (n=768) FCM concentrations (after being standardised for sex-specific variance). Standard errors are in parentheses. This model was a significantly better fit than a model in which among-individual variances were constrained to be the same in both sexes (χ^2^_(1)_=3.583; p=0.007).*

| **(a) Among-individual** | | |  | **(b) Within-individual** | | |
| --- | --- | --- | --- | --- | --- | --- |
|  | M | F |  |  | M | F |
| M | 0.210 (0.103) |  |  | M | 0.792 (0.103) |  |
| F |  | 0.005 (0.021) |  | F |  | 0.995 (0.051) |
